# An integrated molecular, phenotypic, and adaptive analysis of polymyxin resistance in KPC-producing *Klebsiella pneumoniae*

**DOI:** 10.1007/s11274-026-05240-1

**Published:** 2026-11-11

**Authors:** Anelise Stella Ballaben, Joseane Cristina Ferreira, Ludmilla Tonani Carvalho, Doroti de Oliveira Garcia, Robert K. Ernst, Marcia R. von Zeska Kress, Yohei Doi, Ana Lúcia da Costa Darini

**Affiliations:** 1 Department of Agricultural and Environmental Biotechnology, Faculty of Agricultural and Veterinary Sciences, Sao Paulo State University (UNESP), Jaboticabal, Sao Paulo 14884-900 Brazil; 2 School of Pharmaceutical Sciences of Ribeirao Preto, University of Sao Paulo, Ribeirao Preto, Sao Paulo Brazil; 3 Center of Regional Laboratory, Adolfo Lutz Institute, Marilia, Sao Paulo Brazil; 4 Department of Microbial Pathogenesis, School of Dentistry, University of Maryland Baltimore, Baltimore, USA; 5 Center for Innovative Antimicrobial Therapy, Division of Infectious Diseases, University of Pittsburgh School of Medicine, Pittsburgh, PA USA

**Keywords:** Lipid A modifications, PmrAB/PhoPQ TCS, MALDI-TOF-MS, KPC-producing *Klebsiella pneumoniae*

## Abstract

Rising resistance to polymyxins poses a critical challenge in the management of multidrug-resistant (MDR) and extensively drug-resistant (XDR) *Klebsiella pneumoniae*. This study investigated 50 polymyxin-resistant *K. pneumoniae* isolates recovered from hospitals in Brazil using an integrated approach combining antimicrobial susceptibility testing, targeted molecular analysis, lipid A profiling, and phenotypic assays. Eighteen isolates were classified as XDR and 32 as MDR. All isolates carried *bla*_KPC_, 56% co-harbored *bla*_CTX−M−type_ genes, and 62% were resistant to ceftazidime–avibactam. Targeted sequencing revealed diverse alterations in polymyxin resistance-associated loci, particularly *mgrB*,* pmrAB*,* phoQ*, and *crrB*, including amino acid substitutions, premature stop codons, insertions, and deletions. Lipid A analysis by MALDI-TOF MS confirmed structural remodeling, such as the incorporation of 4-amino-4-deoxy-L-arabinose (L-Ara4N) and phosphoethanolamine, consistent with polymyxin-resistant phenotypes. Virulence assessment using the *Galleria mellonella* model showed marked heterogeneity among isolates, with no direct association between resistance-associated alterations and virulence phenotypes. Short-term survival following polymyxin B exposure at pH 5.8 and 7.7 differed in only a subset of isolates, indicating a strain-dependent response to environmental conditions. In addition, the laboratory-evolved *Escherichia coli* D12 mutant exhibited lipid A remodeling and alterations in PmrAB following polymyxin selective pressure. Overall, these findings support the multifactorial nature of polymyxin resistance and highlight the value of integrating molecular, structural, and phenotypic approaches for antimicrobial resistance surveillance.

**Supplementary information:**

The online version contains supplementary material available at https://doi.org/10.1007/s11274-026-05240-1.

## Introduction

The increasing resistance to antibiotics, particularly polymyxins, has become one of the most pressing public health concerns (Ho et al. 2024). Polymyxins bind to the negatively charged phosphate groups of lipid A, a key component of lipopolysaccharide (LPS), displacing divalent cations such as Ca²⁺ and Mg²⁺. This interaction destabilizes the LPS, leading to increased membrane permeability, leakage of cytoplasmic contents, and ultimately bacterial cell death (Doi and van Duin 2020). Initially discovered in 1947 and used briefly in clinical practice, polymyxins were largely replaced due to their high nephrotoxicity and neurotoxicity. However, with the rise of multidrug resistance (MDR) and extensively drug-resistant (XDR) in Gram-negative bacteria, polymyxins have been revived as last-resort treatments for infections resistant to all other available antibiotics (Doi and van Duin 2020).

Despite their critical role in treating MDR/XDR infections, the emergence of polymyxin resistance has severely compromised their utility. Resistance to polymyxins mainly involves modifications to lipid A (Olaitan et al. 2014; Nang et al. 2019). The most common modifications include the addition of the cationic sugars 4-amino-L-arabinose (L-Ara4N) and/or phosphoethanolamine (PEtN), which reduce the net negative charge of lipid A and prevent effective binding by polymyxins. These structural changes vary among different bacterial species and follow distinct mechanisms depending on whether the resistance is plasmid- or chromosome-encoded (Nang et al. 2019; Doi and van Duin 2020).

For most Gram-negative bacteria, resistance is acquired through mutations or horizontal gene transfer. Chromosome-encoded polymyxin resistance typically involves alterations in regulatory pathways controlling lipid A remodeling, particularly the *PmrA*/*PmrB*, *PhoP*/*PhoQ*, and CrrA/*CrrB* two-component systems (TCS) and the negative regulator *MgrB*. Dysregulation of these pathways can activate downstream genes involved in lipid A modification, thereby reducing the affinity of polymyxins for the bacterial outer membrane (Huang et al. 2020; Sun et al. 2022).

In addition to lipid A modification, other mechanisms contribute to polymyxin resistance. These include the expression of efflux pumps, which actively expel polymyxins from the bacterial cell, capsule formation that may protect the bacteria from the drug’s effects, and the overexpression of outer membrane proteins like OprH. Together, these mechanisms enhance the ability of bacteria to survive in the presence of polymyxin antibiotics (Doi and van Duin 2020).

Recently, an epidemiological review in the Americas highlighted the alarming spread of carbapenem-resistant *Klebsiella* pathogens that produce carbapenemases including KPC and NDM and are also resistant to polymyxins (Rodríguez-Santiago et al. 2021).

This study adopted a targeted molecular strategy focusing on canonical polymyxin resistance pathways previously established in *K. pneumoniae*, integrated with phenotypic, lipid A, and environmental modulation analyses.

The primary aim of this study was to characterize the antimicrobial resistance profiles and candidate genetic alterations associated with polymyxin resistance in contemporary KPC-producing *Klebsiella pneumoniae* clinical isolates from Brazil. Secondary aims were: (i) to assess lipid A structural features by MALDI-TOF-MS; (ii) to evaluate virulence potential in the *Galleria mellonella* infection model; and (iii) to explore the influence of environmental pH on short-term survival following polymyxin B exposure in a subset of isolates.

## Materials and methods

### Clinical isolates, species identification, and ethics

Fifty polymyxin-resistant *Klebsiella* spp. isolates were obtained from 22 hospitals across São Paulo State between 2016 and 2022. They were recovered from diverse clinical specimens and referred to the Adolfo Lutz Institute Public Health Laboratory (Marília, Brazil) for further investigation. Species identification was performed by MALDI-TOF MS using the MALDI TypeR platform from the Pasteur Institute (https://maldityper.pasteur.fr/).

The study was approved by the Ethics Committee of the School of Pharmaceutical Sciences of Ribeirão Preto, University of São Paulo (approval no. 20413419.2.0000.5403).

### Antimicrobial susceptibility testing

Antimicrobial susceptibility testing (AST) was performed by disk diffusion on Mueller-Hinton Agar (Oxoid, Ltd, Basingstoke, UK) and the results were interpreted in accordance with the recommendations of the Brazilian Committee on Antimicrobial Susceptibility Testing (BrCAST). Twenty-nine antibiotics were tested: amoxicillin/clavulanate (20/10 µg), cefotaxime (30 µg), cefpodoxime (30 µg), ceftazidime (30 µg), cefepime (30 µg), ceftaroline (30 µg), cefoxitin (30 µg), ceftazidime-avibactam (30/20 µg), aztreonam (30 µg), ertapenem (10 µg), meropenem (10 µg), imipenem (10 µg), doripenem (10 µg), gentamicin (10 µg), tobramycin (10 µg), amikacin (30 µg), ciprofloxacin (5 µg), levofloxacin (5 µg), nalidixic acid (30 µg), norfloxacin (10 µg), tetracycline (30 µg), minocycline (30 µg), doxycycline (30 µg), nitrofurantoin (300 µg), piperacillin-tazobactam (100/10 µg), ticarcillin-clavulanate (75/10 µg), ampicillin-sulbactam (10/10 µg), chloramphenicol (30 µg), and trimethoprim-sulfamethoxazole (1.25/23.75 µg). Minimum inhibitory concentrations (MICs) were determined for cefotaxime, ceftazidime, imipenem, meropenem, polymyxin B, tigecycline, and fosfomycin by Etest and/or by broth microdilution according to the BrCAST and/or CLSI guidelines. According to the standard definitions, the isolates were classified as MDR, XDR, and pandrug-resistant (PDR) (Magiorakos et al. 2012).

### Detection and sequence analysis of antimicrobial resistance and polymyxin resistance-associated genes

PCR was used to investigate ARGs coding for β-lactamases (*bla*_CTX−like_, *bla*_TEM_, *bla*_SHV_, *bla*_CMY_, *bla*_KPC_, *bla*_IMP_, *bla*_NDM_, *bla*_VIM_, *bla*_OXA−48_), tetracycline resistance determinants, plasmid-mediated quinolone resistance determinants, 16S rRNA methyltransferases, plasmid-mediated colistin resistance genes (*mcr-1* to *mcr-5*), and chromosomal polymyxin resistance-associated loci (*pmrA*,* pmrB*,* phoP*,* phoQ*,* mgrB*,* crrB*) using previously described primers (Esposito et al. 2018; Rebelo et al. 2018; Ballaben et al. 2022). All *bla*_KPC_-positive amplicons were subjected to sequencing, and KPC alleles were assigned by comparison of the obtained nucleotide sequences with reference sequences deposited in the NCBI National Database of Antibiotic-Resistant Organisms (NDARO). The nucleotide sequences of *pmrA*,* pmrB*,* phoP*,* phoQ*,* mgrB*, and *crrB* were determined by Sanger sequencing and compared with the corresponding loci from *Klebsiella pneumoniae subsp. pneumoniae* HS11286 (GenBank accession CP003200.1). Deduced amino acid sequences were subsequently compared with the corresponding reference proteins to identify amino acid substitutions, premature stop codons, insertions, and deletions.

### Lipid A profiling by MALDI-TOF MS

Lipid A profiles of the *K. pneumoniae* isolates were analyzed by matrix-assisted laser desorption/ionization time-of-flight mass spectrometry (MALDI-TOF MS) in negative-ion reflectron mode, following the protocol described by Yang et al. (2022). Mass spectra were acquired and processed using flexControl and flexAnalysis software (v3.4, Bruker Daltonics Inc.). Lipid A-associated ions were evaluated for the presence of structural modifications related to polymyxin resistance, including L-Ara4N and phosphoethanolamine (PEtN) incorporation, palmitoylation, and hydroxylation.

### *In vivo* virulence evaluation in *Galleria mellonella*

The *Galleria mellonella* infection model was employed to assess the virulence of *K. pneumoniae* isolates and *Escherichia coli* ATCC 25922, following established protocols (Yang et al. 2018; Paziani et al. 2019). Groups of 10 larvae at the sixth instar (225 ± 50 mg, without cuticle pigmentation) were injected with 10 µL of inoculum into the hemocoel through the last right proleg using a Hamilton syringe. Inoculum consisted of either PBS 1× (negative control) or bacterial suspensions adjusted to 1.5 × 10⁸ CFU/mL. After injection, larvae were incubated in glass containers at 37 °C in the dark and monitored daily for survival for up to eight days.

For descriptive comparison, isolates were categorized according to the survival profile observed in the *G. mellonella* assay: isolates associated with 90–100% larval survival at the end of the experiment were classified as low/non-virulent (Group A), those associated with approximately 30–60% survival were classified as moderately virulent (Group B), and isolates causing complete larval mortality during the observation period were classified as highly virulent (Group C). These categories were used to describe the relative virulence phenotypes observed under experimental conditions and do not represent validated clinical definitions of hypervirulence.

Survival curves were plotted using the Kaplan–Meier method, and statistical significance was evaluated by the log-rank (Mantel–Cox) test in GraphPad Prism v5.0, with *P *< 0.05 considered significant.

### Short-term survival following polymyxin B exposure under different pH conditions

*Klebsiella pneumoniae* isolates carrying amino acid changes in *PmrA*, *PmrB*, *PhoP*, or *PhoQ* were evaluated for short-term survival following polymyxin B exposure under acidic (pH 5.8) and comparative reference (pH 7.7) conditions. The assay was adapted from the 1-h survival experimental framework described by Adams et al. (2009). Briefly, isolates were cultured in lysogeny broth (LB) adjusted to pH 5.8 or 7.7 for 3–4 h at 37 °C with shaking. For each isolate and pH condition, parallel cultures were either maintained without polymyxin B as growth controls or exposed to polymyxin B at a concentration corresponding to 10× the previously determined MIC for that isolate. After 1 h of polymyxin B exposure, serial dilutions were prepared and plated onto Mueller–Hinton agar. Plates were incubated for 18–24 h at 37 °C, and viable cells were quantified by colony-forming unit (CFU) counts. Short-term survival was assessed based on the recovery of viable cells following polymyxin B exposure relative to the corresponding culture maintained without polymyxin B at the same pH. *K. pneumoniae* ATCC 13883 was included as the experimental control.

### Transcriptional analysis by qRT-PCR

qRT-PCR was performed on selected clinical isolates carrying amino acid alterations in polymyxin resistance-associated regulatory proteins. The *pmrA*,* pmrB*,* pmrD*,* pmrE*,* pmrK*, and *phoQ* genes were selected based on their roles in the PmrAB- and PhoPQ-associated regulatory pathways (Cannatelli et al. 2013; Jayol et al. 2014). For RNA extraction, isolates were regrown under conditions informed by the phenotypes observed in the short-term survival assay. Isolates in which viable cells were recovered after the polymyxin B challenge under a specific pH condition were regrown in lysogeny broth (LB) adjusted to the corresponding pH. Isolates for which no viable cells were recovered after the polymyxin B challenge under either pH condition were regrown in LB without adjustment to a specific experimental pH. Cultures were incubated for 12 h at 37 °C with shaking at 175 rpm and subsequently adjusted to a turbidity corresponding to McFarland 2. Following regrowth, cultures were exposed again to polymyxin B at 10× the previously determined MIC for each isolate, matching the concentration used in the short-term survival assay.

Bacterial RNA was stabilized using RNAprotect Bacteria Reagent (Qiagen) and purified using the RNeasy Mini Kit (Qiagen), according to the manufacturer’s instructions. RNA samples were treated with DNase prior to cDNA synthesis. qRT-PCR measurements were performed in technical duplicate using the same RNA preparation for each isolate. Relative gene expression was calculated using the comparative Ct (2^-ΔΔCt) method (Livak and Schmittgen 2001), with expression normalized to the *rpsL* housekeeping gene and *K. pneumoniae* ATCC 700603 (polymyxin B MIC, 0.5 µg/mL) used as the experimental calibrator.

### Polymyxin B selective-pressure experiment with *E. coli* ATCC 25922

*E. coli* ATCC 25922 was evaluated over a 12-day experiment under selective pressure, using lysogenic broth with polymyxin B concentrations ranging from 0.125 to 256 µg/mL, incubated at 37 °C with shaking. Aliquots of 1 mL were taken at days 0 (WT), 4, 8, and 12, and stored at -80 °C for further analysis.

### Whole-genome sequencing and in silico analysis of *E. coli* D12

WGS was performed using the Illumina MiSeq platform with paired-end reads (2 × 250 bp) and an estimated sequencing depth of approximately 155× for the *E. coli* ATCC 25922 mutant recovered on day 12 (*E. coli* D12) of the polymyxin B selective-pressure experiment. Paired-end reads were assembled using SPAdes v4.0.0 (Bankevich et al. 2012) in standard mode, with automatic *k*-mer selection and read error correction. Assembly quality was assessed using QUAST v5.3.0 (Gurevich et al. 2013), with the main assembly statistics calculated considering contigs ≥ 500 bp. The genome was annotated using Bakta v1.10.1 (Schwengers et al. 2021), and genome completeness and contamination were evaluated using CheckM v1.2.2 (Parks et al. 2015).

## Results

### Antimicrobial resistance and ARG profiles

All isolates were confirmed as *K. pneumoniae* by MALDI-TOF MS and the MALDI TypeR platform and were recovered from urine (*n* = 19), blood (*n* = 16), tracheal secretion (*n* = 6), rectal swab (*n* = 6), catheter tip (*n* = 1), peritoneal fluid (*n* = 1), and unknown (*n* = 1). Eighteen of the 50 isolates were classified as XDR, while the remaining 32 were categorized as MDR. Among the tested antimicrobials, aminoglycosides exhibited the highest number of susceptible isolates. All 50 isolates (100%) carried *bla*_KPC_, and 28 (56%) also carried *bla*_CTX-M_ genes belonging to groups 1, 2, or 9. Moreover, 39 (78%) isolates presented *tetA* and 6 of them also carried *tetB*; plasmid-mediated quinolone resistance genes, *qnrS* and *qnrB* were found in 6 and 1 isolates, respectively. All the isolates that presented a high-level aminoglycoside resistance phenotype produced *rmtB* (*n* = 18). Additionally, since all isolates were KPC producers, ceftazidime–avibactam (CAZ-AVI) susceptibility was tested, and 31 out of 50 isolates (62%) were resistant (Table 1). Sequencing of *bla*_KPC_ allowed allele assignment in 42 of the 50 isolates (84%), all of which were identified as *bla*_KPC−2_. An unambiguous KPC allele could not be assigned to the remaining eight isolates, which were therefore designated *bla*_KPC−like_. Of these, six isolates (Kp 184/18, Kp 27/21, Kp 72/21, Kp 149/21, Kp 34/22, and Kp 288/22) were resistant to CAZ-AVI, whereas two (Kp 237/16 and Kp 47/22) were susceptible (Table 1). The correlation between phenotypic and genotypic profiles was consistent, except for isolate Kp 47/22, which remained phenotypically susceptible to imipenem and meropenem (MIC 0.38 and 1 µg/mL, respectively) despite carrying the *bla*_KPC_ gene.

**Table 1:** Phenotypic, molecular, and virulence characteristics of the 50 polymyxin B-resistant *Klebsiella pneumoniae* clinical isolates included in the study

Isolate/Year (Source)	Resistances ^a^	MIC (µg/mL) ^b^							Resistance-associated genes/loci	Virulence profile
		CTX	CAZ	IPM	MER	PMB	TIG	FOS		
48/22 (urine) #	SAM, TZP, TIM, CTX, CAZ, CPO, FEP, CPT, ATM, AMC, ETP, IPM, MER, DOR, GEN, TOB, AMK, TET, NA, CIP, LEV, SXT, CHL	≥ 256	16	≥ 256	≥ 32	16	≥ 1	ND	*bla*_KPC−2_, *tetA*,* rmtB*,* mgrB*,* crrB*	High virulence
292/22 (urine) #	SAM, TZP, TIM, CTX, CAZ, CPO, FEP, CPT, ATM, AMC, ETP, MER, DOR, GEN, TOB, AMK, TET, NA, CIP, LEV, SXT, CHL	≥ 256	48	8	16	32	1	ND	*bla*_KPC−2_, *bla*_CTX−M−group 1_, *tetA*,* rmtB*,* crrB*	
339/22 (urine) #	SAM, TZP, TIM, CTX, CAZ, CPO, FEP, CPT, CZA, ATM, AMC, ETP, IPM, MER, DOR, GEN, TOB, AMK, TET, NA, CIP, LEV, SXT, CHL	≥ 256	32	64	≥ 32	16	1	4	*bla*_KPC−2_, *bla*_CTX−M−group 9_, *tetA*,* rmtB*,* mgrB*,* crrB*	
276/21 (urine)	SAM, TZP, TIM, CTX, CAZ, CPO, FEP, CPT, ATM, AMC, ETP, IPM, MER, DOR, GEN, TOB, AMK, TET, NA, CIP, LEV, SXT, CHL	≥ 256	18	64	≥ 32	32	1	12	*bla*_KPC−2_, *bla*_CTX−M−group 1_, *tetA*,* rmtB*,* mgrB*,* crrB*	
909/16 (urine)	SAM, ATM, CTX, CPO, CAZ, FEP, CPT, CZA, ETP, IPM, MER, NA, CIP, NOR, AMK, TOB, TZP, SXT, TIM, TET, NIT	≥ 256	64	> 32	> 32	16	1,0	ND	*bla*_KPC−2_, *bla*_CTX−M group 1_, *mgrB*,* crrB*	
346/16 (urine)	SAM, ATM, CTX, CPO, CAZ, FEP, CPT, ETP, IPM, MER, NA, CIP, NOR, AMK, GEN, TOB, TZP, SXT, TIM, TET, NIT	≥ 256	64	> 32	> 32	48	1	ND	*bla*_KPC−2_, *bla*_CTX−M group 2_, *mgrB*,* crrB*	
288/17 (urine)	SAM, ATM, CTX, CPO, CAZ, FEP, CPT, CZA, ETP, IPM, MER, NA, CIP, NOR, GEN, TZP, SXT, TIM, TET, NIT	> 256	> 256	> 32	> 32	32	ND	ND	*bla*_KPC−2_, *mgrB*,* crrB*	
192/22 (urine)	SAM, TZP, TIM, CTX, CAZ, CPO, FEP, CPT, ATM, AMC, ETP, IPM, MER, DOR, TOB, AMK, TET, NA, CIP, LEV, SXT, CHL	≥ 256	24	≥ 256	≥ 32	16	2	ND	*bla*_KPC−2_, *tetA*,* crrB*	
58/16 (urine)	SAM, ATM, CTX, CPO, CAZ, FEP, CPT, CZA, ETP, IPM, MER, NA, CIP, NOR, GEN, TOB, SXT, TIM, TET, NIT	≥ 256	> 256	32	> 32	12	1,5	ND	*bla*_KPC−2_, *bla*_CTX−M group 2_, *mgrB*,* crrB*	Moderate virulence
983/16 (urine)	SAM, ATM, CTX, CPO, CAZ, FEP, CPT, CZA, ETP, IPM, MER, NA, CIP, NOR, TOB, TZP, SXT, TIM, NIT	≥ 256	64	> 32	> 32	16	1,5	ND	*bla*_KPC−2_, *bla*_CTX−M group 1_, *mgrB*,* crrB*	
10/21(urine)	SAM, TZP, TIM, CTX, CAZ, CPO, FEP, CPT, ATM, AMC, ETP, IPM, MER, DOR, GEN, TOB, AMK, TET, NA, CIP, LEV, SXT, CHL	≥ 256	24	48	≥ 32	64	1	8	*bla*_KPC−2_, *tetA*,* rmtB*,* mgrB*,* crrB*	
27/21 (urine)	SAM, TZP, TIM, CTX, CAZ, CPO, FEP, CPT, CZA, ATM, AMC, ETP, IPM, MER, DOR, GEN, TOB, AMK, TET, DOX, NA, CIP, LEV, NOR, SXT, CHL, NIT	≥ 256	≥ 256	32	4	16	≥ 2	12	*bla*_KPC−like_, *qnrS*,* tetA*,* tetB*,* mgrB*,* crrB*	
640/21 (urine)	SAM, TZP, TIM, CTX, CAZ, CPO, FEP, CPT, ATM, AMC, ETP, IPM, MER, DOR, GEN, TOB, AMK, TET, NA, CIP, LEV, SXT, CHL	≥ 256	48	≥ 256	≥ 32	32	0,5	ND	*bla*_KPC−2_, *tetA*,* rmtB*,* mgrB*,* crrB*	
426/21 (urine)	SAM, TZP, TIM, CTX, CAZ, CPO, FEP, CPT, ATM, AMC, ETP, IPM, MER, DOR, TOB, TET, NA, CIP, LEV, SXT, CHL	≥ 256	48	≥ 256	≥ 32	32	≥ 2	ND	*bla*_KPC−2_, *bla*_CTX−M−group 1_, *tetA*,* mgrB*,* crrB*	
38/22 (urine)	SAM, TZP, TIM, CTX, CAZ, CPO, FEP, CPT, ATM, AMC, ETP, IPM, MER, DOR, GEN, TOB, AMK, TET, NA, CIP, LEV, SXT, CHL	≥ 256	32	≥ 256	≥ 32	≥ 64	≥ 2	16	*bla*_KPC−2_, *bla*_CTX−M−group 1_, *tetA*,* rmtB*,* crrB*	
47/22 (urine)	SAM, TZP, TIM, CTX, CAZ, CPO, FEP, CPT, ATM, AMC, ETP, GEN, TOB, AMK, TET, NA, CIP, LEV, SXT, CHL	≥ 256	8	0.38	1	16	≥ 1	ND	*bla*_KPC−like_, *tetA*,* rmtB*,* mgrB*,* crrB*	
790/16 (urine)	SAM, ATM, CTX, CPO, CAZ, FEP, CPT, CZA, ETP, IPM, MER, CIP, TOB, TZP, SXT, TIM, TET	≥ 256	> 256	12	> 32	48	0,5	ND	*bla*_KPC−2_, *bla*_CTX−M group 1_, *mgrB*,* crrB*	
429/16 (urine)	SAM, ATM, CTX, CPO, CAZ, FEP, CPT, CZA, ETP, IPM, MER, NA, CIP, NOR, TZP, TIM	≥ 256	64	0,38	1	16	0,19	ND	*bla*_KPC−2_, *mgrB*,* crrB*	Low/non-virulent
279/17 (urine)	SAM, ATM, CTX, CPO, CAZ, FEP, CPT, CZA, ETP, IPM, MER, NA, CIP, TZP, SXT, TIM, TET	> 256	192	12	6	3	1	ND	*bla*_KPC−2_, *bla*_CTX−M group 1_, *qnrS*,* mgrB*,* crrB*	
1013/16 (blood)	SAM, ATM, CTX, CPO, CAZ, FEP, CPT, ETP, IPM, MER, CIP, AMK, TOB, TZP, TIM, TET, CHL	≥ 256	64	2	0,38	16	0,75	ND	*bla*_KPC−2_, *mgrB*,* crrB*	High virulence
322/17 (blood)	SAM, ATM, CTX, CPO, CAZ, FEP, CPT, CZA, ETP, IPM, MER, CIP, AMK, TOB, TZP, SXT, TIM, TET, CHL	> 256	> 256	> 32	> 32	32	1,5	ND	*bla*_KPC−2_, *bla*_CTX−M group 1_, *mgrB*,* crrB*	
388/16 (blood)	SAM, ATM, CTX, CPO, CAZ, FEP, CPT, CZA, ETP, IPM, MER, CIP, TZP, TIM	≥ 256	> 256	> 32	> 32	32	0,38	ND	*bla*_KPC−2_, *mgrB*,* crrB*	
100/17 (blood)	SAM, ATM, CTX, CPO, CAZ, FEP, CPT, CZA, ETP, IPM, MER, CIP, TZP, TIM	> 256	16	32	> 32	16	1	ND	*bla*_KPC−2_, *mgrB*,* crrB*	
110/17 (blood)	SAM, ATM, CTX, CPO, CAZ, FEP, CPT, CZA, ETP, IPM, MER, CIP, TZP, SXT, TIM, TET	32	24	24	> 32	48	0,75	ND	*bla*_KPC−2_, *mgrB*,* crrB*	
48/21 (blood) #	SAM, TZP, TIM, CTX, CAZ, CPO, FEP, CPT, CZA, ATM, AMC, ETP, IPM, MER, DOR, GEN, TOB, AMK, TET, NA, CIP, LEV, SXT, CHL	≥ 256	32	≥ 256	≥ 32	16	1	ND	*bla*_KPC−2_, *tetA*,* tetB*,* rmtB*,* mgrB*,* crrB*	Moderate virulence
18/22 (blood) #	SAM, TZP, TIM, CTX, CAZ, CPO, FEP, CPT, CZA, ATM, AMC, ETP, IPM, MER, DOR, GEN, TOB, AMK, TET, NA, CIP, LEV, SXT, CHL	≥ 256	32	≥ 256	≥ 32	64	≥ 2	16	*bla*_KPC−2_, *tetA*,* rmtB*,* mgrB*,* crrB*	
209/22 (blood) #	SAM, TZP, TIM, CTX, CAZ, CPO, FEP, CPT, ATM, AMC, ETP, IPM, MER, DOR, GEN, TOB, AMK, TET, NA, CIP, LEV, SXT, CHL	≥ 256	32	≥ 256	≥ 32	32	≥ 1	16	*bla*_KPC−2_, *tetA*,* rmtB*,* crrB*	
182/16 (blood)	SAM, ATM, CTX, CPO, CAZ, FEP, CPT, ETP, IPM, MER, AMK, GEN, TOB, TZP, SXT, TIM, CHL	≥ 256	64	32	> 32	12	1,5	ND	*bla*_KPC−2_, *bla*_CTX−M group 2_, *mgrB*,* crrB*	
184/18 (blood)	SAM, ATM, CTX, CPO, CAZ, FEP, CPT, CZA, ETP, IPM, MER, CIP, LEV, TOB, TZP, SXT, TIM, CHL	> 256	> 256	> 32	> 32	32	0,75	ND	*bla*_KPC−like_, *bla*_CTX−M group 2_, *mgrB*,* crrB*	
185/21 (blood)	SAM, TZP, TIM, CTX, CAZ, CPO, FEP, CPT, ATM, AMC, ETP, IPM, MER, DOR, TOB, AMK, TET, NA, CIP, LEV, SXT	≥ 256	≥ 256	≥ 256	≥ 32	16	≥ 2	ND	*bla*_KPC−2_, *bla*_CTX−M−group 1_, *tetA*,* mgrB*	
281/22 (blood)	SAM, TZP, TIM, CTX, CAZ, CPO, FEP, CPT, ATM, AMC, ETP, IPM, MER, DOR, TET, NA, CIP, LEV, SXT, CHL	≥ 256	24	16	12	16	2	ND	*bla*_KPC−2_, *bla*_CTX−M−group 1_, *tetA*,* crrB*	
641/21 (blood)	SAM, TZP, TIM, CTX, CAZ, CPO, FEP, CPT, CZA, ATM, AMC, ETP, IPM, MER, DOR, TOB, AMK, TET, NA, CIP, SXT, CHL	≥ 256	≥ 256	48	≥ 32	16	0,5	ND	*bla*_KPC−2_, *tetA*,* mgrB*	
237/16 (blood)	SAM, ATM, CTX, CPO, CAZ, FEP, CPT, ETP, IPM, MER, CIP, TZP, SXT, TIM, CHL	≥ 256	64	12	4	48	1,5	ND	*bla*_KPC−like_, *bla*_CTX−M group 2_, *mgrB*,* crrB*	Low/non-virulent
480/17 (blood)	SAM, ATM, CTX, CPO, CAZ, FEP, CPT, CZA, ETP, IPM, MER, CIP, TOB, TZP, TIM	> 256	> 256	> 32	> 32	64	1	ND	*bla*_KPC−2_, *qnrS*,* mgrB*,* crrB*	
172/17 (blood)	SAM, ATM, FOX, CTX, CPO, CAZ, FEP, CPT, ETP, IPM, MER, CIP, LEV, TOB, TZP, SXT, TIM, CHL	≥ 256	> 256	> 32	4	64	ND	ND	*bla*_KPC−2_, *mgrB*,* crrB*	
114/21 (tracheal secretion) #	SAM, TZP, TIM, CTX, CAZ, CPO, FEP, CPT, ATM, AMC, ETP, IPM, MER, DOR, GEN, TOB, AMK, TET, NA, CIP, LEV, SXT, CHL	≥ 256	16	64	≥ 32	32	1	32	*bla*_KPC−2_, *bla*_CTX−M−group 1_, *tetA*,* tetB*,* rmtB*,* mgrB*,* crrB*	High virulence
247/21 (tracheal secretion) #	SAM, TZP, TIM, CTX, CAZ, CPO, FEP, CPT, ATM, AMC, ETP, IPM, MER, DOR, GEN, TOB, AMK, TET, NA, CIP, LEV, SXT, CHL	≥ 256	≥ 256	≥ 256	≥ 32	8	≥ 1	ND	*bla*_KPC−2_, *bla*_CTX−M−group 1_, *tetA*,* rmtB*,* mgrB*,* crrB*	
335/21 (tracheal secretion) #	SAM, TZP, TIM, CTX, CAZ, CPO, FEP, CPT, ATM, AMC, ETP, IPM, MER, DOR, GEN, TOB, AMK, TET, DOX, NA, CIP, LEV, SXT, CHL	≥ 256	64	64	≥ 32	64	≥ 1	32	*bla*_KPC−2_, *bla*_CTX−M−group 1_, *tetA*,* rmtB*,* mgrB*,* crrB*	
593/21 (tracheal secretion) #	SAM, TZP, TIM, CTX, CAZ, CPO, FEP, CPT, CZA, ATM, AMC, ETP, IPM, MER, DOR, GEN, TOB, AMK, TET, NA, CIP, LEV, SXT, CHL	≥ 256	18	≥ 256	≥ 32	64	1	6	*bla*_KPC−2_, *bla*_CTX−M−group 1_, *tetA*,* rmtB*,* mgrB*,* crrB*	
34/22 (tracheal secretion)	SAM, TZP, TIM, CTX, CAZ, CPO, FEP, CPT, CZA, ATM, AMC, ETP, IPM, MER, DOR, GEN, TOB, TET, NA, CIP, LEV, SXT	≥ 256	≥ 256	24	≥ 32	64	2	ND	*bla*_KPC−like_, *bla*_CTX−M−group 1_, *tetA*,* qnrS*,* mgrB*,* crrB*	Low/non-virulent
216/22 (tracheal secretion)	SAM, TZP, TIM, CTX, CAZ, CPO, FEP, CPT, ATM, AMC, ETP, IPM, MER, DOR, GEN, TOB, AMK, TET, NA, CIP, LEV, SXT, CHL	≥ 256	32	24	≥ 32	8	1	ND	*bla*_KPC−2_, *bla*_CTX−M−group 1_, *tetA*,* qnrS*	
324/22 (rectal swab) #	SAM, TZP, TIM, CTX, CAZ, CPO, FEP, CPT, ATM, AMC, ETP, IPM, MER, DOR, GEN, TOB, AMK, TET, NA, CIP, LEV, SXT, CHL	≥ 256	32	≥ 256	≥ 32	64	≥ 2	8	*bla*_KPC−2_, *bla*_CTX−M−group 1_, *tetA*,* tetB*,* rmtB*,* mgrB*,* crrB*	High virulence
36/22 (rectal swab) #	SAM, TZP, TIM, CTX, CAZ, CPO, FEP, CPT, ATM, AMC, ETP, IPM, MER, DOR, GEN, TOB, AMK, TET, NA, CIP, LEV, SXT, CHL	≥ 256	12	64	≥ 32	64	1	6	*bla*_KPC−2_, *bla*_CTX−M−group 9_, *tetA*,* rmtB*,* mgrB*,* crrB*	
230/22 (rectal swab) #	SAM, TZP, TIM, CTX, CAZ, CPO, FEP, CPT, ATM, AMC, ETP, IPM, MER, DOR, GEN, TOB, AMK, TET, NA, CIP, LEV, SXT, CHL	≥ 256	32	≥ 256	≥ 32	4	≥ 1	≥ 1024	*bla*_KPC−2_, *bla*_CTX−M−group 1_, *tetA*,* mgrB*	
288/22 (rectal swab)	SAM, TZP, TIM, CTX, CAZ, CPO, FEP, CPT, CZA, ATM, AMC, ETP, IPM, MER, DOR, TET, NA, CIP, LEV, SXT, CHL	≥ 256	≥ 256	4	8	4	≥ 1	ND	*bla*_KPC−like_, *tetA*,* qnrS*,* mgrB*,* crrB*	
259/22 (rectal swab)	SAM, TZP, TIM, CTX, CAZ, CPO, FEP, CPT, ATM, AMC, ETP, IPM, MER, DOR, TOB, TET, NA, CIP, LEV, SXT, CHL	≥ 256	≥ 256	≥ 256	≥ 32	16	1	ND	*bla*_KPC−2_, *tetA*,* mgrB*,* crrB*	
11/22 (rectal swab) #	SAM, TZP, TIM, CTX, CAZ, CPO, FEP, CPT, ATM, AMC, ETP, IPM, MER, DOR, GEN, TOB, AMK, TET, NA, CIP, LEV, SXT, CHL	≥ 256	48	≥ 256	≥ 32	64	≥ 2	16	*bla*_KPC−2_, *bla*_CTX−M−group 1_, *tetA*,* rmtB*,* mgrB*,* crrB*	Low/non-virulent
256/22 (peritoneal fluid) #	SAM, TZP, TIM, CTX, CAZ, CPO, FEP, CPT, CZA, ATM, AMC, ETP, IPM, MER, DOR, GEN, TOB, AMK, TET, NA, CIP, LEV, SXT, CHL	≥ 256	96	≥ 256	≥ 32	4	1	≥ 1024	*bla*_KPC−2_, *bla*_CTX−M−group 1_, *tetA*,* rmtB*,* mgrB*,* crrB*	
72/21 (fragment)	SAM, TZP, TIM, CTX, CAZ, CPO, FEP, CPT, CZA, ATM, AMC, ETP, IPM, MER, DOR, GEN, TOB, AMK, TET, NA, CIP, LEV, SXT	≥ 256	≥ 256	24	4	32	1	ND	*bla*_KPC−like_, *tetA*,* mgrB*,* crrB*	
149/21 (catheter tip)	SAM, TZP, TIM, CTX, CAZ, CPO, FEP, CPT, CZA, ATM, AMC, ETP, IPM, MER, DOR, TET, NA, CIP, LEV, SXT, CHL	≥ 256	≥ 256	≥ 256	≥ 32	64	2	ND	*bla*_KPC−2_, *tetA*,* qnrB*,* crrB*	

# XDR isolates

^a^ SAM sulbactam-ampicillin, TZP piperacillin-tazobactam, TIM ticarcillin-clavulanate, CTX cefotaxime, CAZ ceftazidime, CPO cefpodoxime, FEP cefepime, CPT ceftaroline, CZA ceftazidime-avibactam, ATMaztreonam, AMC amoxicillin-clavulanic acid, ETP ertapenem, IPM imipenem, MERmeropenem, DOR doripenem, GEN gentamicin, TOB tobramycin, AMK amikacin, TET tetracycline, DOX doxycycline, NA nalidixic acid, CIP ciprofloxacin, LEV levofloxacin, NOR norfloxacin, SXT sulfamethoxazole-trimethoprim, CHL chloramphenicol, NIT nitrofurantoin

^b^ The MIC for CTX, CAZ, IPM, and MER was performed using E-test; PMB, polymyxin B; TIG, tigecycline. MICs for PMB and TIG were determined by broth microdilution according to BrCAST recommendations. The BrCAST breakpoints were ≤ 2 µg/mL (susceptible) and > 2 µg/mL (resistant) for PMB, and ≤ 0.5 µg/mL (susceptible) and > 0.5 µg/mL (resistant) for TIG. FOS: fosfomycin; MIC was performed by agar dilution, as prescribed. According to CLSI, the breakpoints for FOS are: ≤ 64 µg/mL (susceptible), ≥ 256 µg/mL (resistant). ND: not determined

### Genetic alterations in TCS-associated loci and *mgrB*

**Fig. 1:**
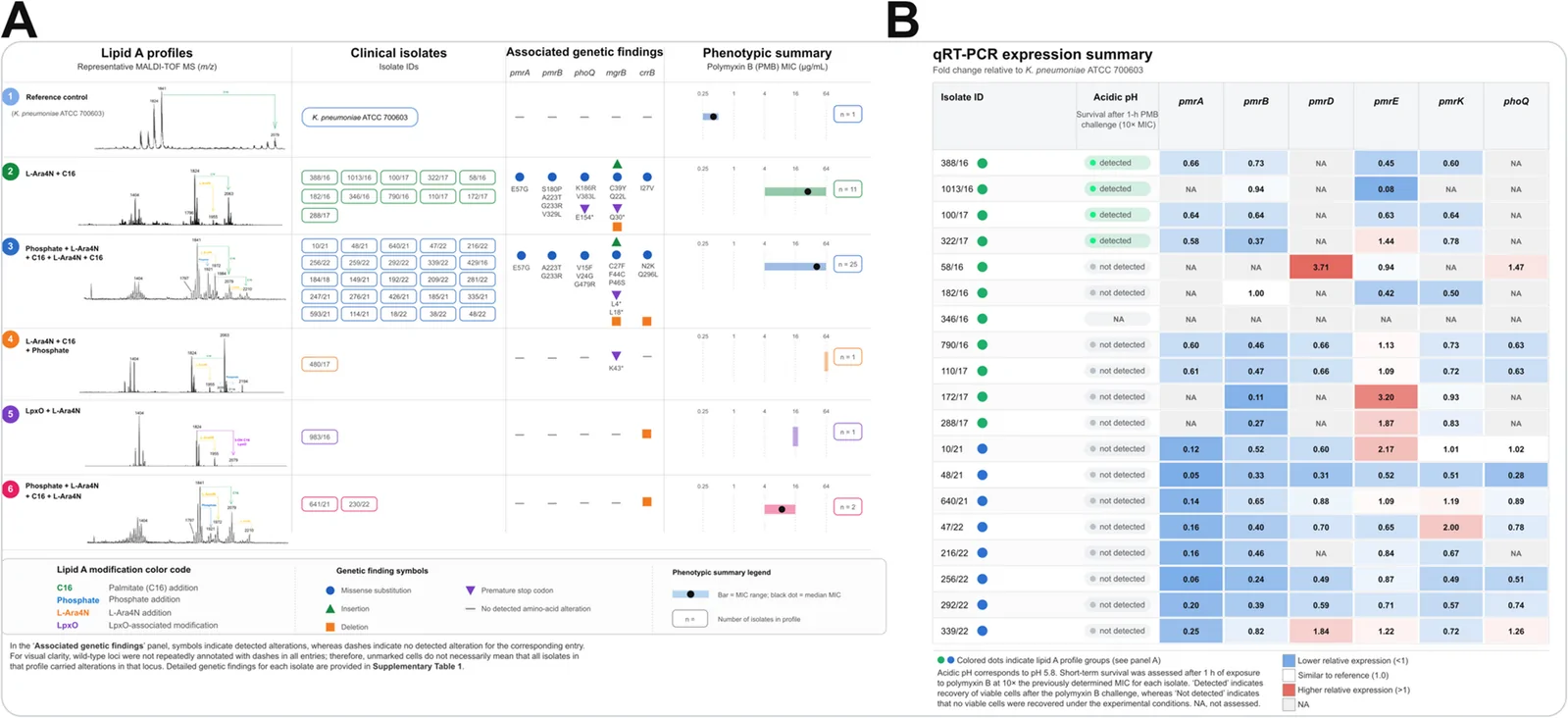
(**A**) Lipid A profiles, associated genetic findings, and polymyxin B MIC distributions among the 40 clinical isolates with detected genetic alterations in the targeted polymyxin resistance-associated loci. A representative MALDI-TOF MS lipid A spectrum is shown for each profile. Profile 1 corresponds to the *K. pneumoniae* ATCC 700603 reference control and is not included among the five lipid A profiles identified in the clinical isolates (profiles 2–6). Clinical isolates sharing the same lipid A profile are grouped together. Lipid A modifications are color-coded as indicated in the figure. Genetic symbols represent missense substitutions, insertions, deletions, and premature stop codons. Dashes indicate no detected amino acid alteration for the corresponding entry. For visual clarity, loci without detected amino acid alterations were not repeatedly marked with dashes across all isolates within each profile; therefore, unmarked entries do not indicate that all isolates in that profile carried an alteration in the corresponding locus. Detailed isolate-level genetic findings are provided in Supplementary Table 1. Polymyxin B MIC bars indicate the range observed within each lipid A profile, and the black dot indicates the median MIC; n indicates the number of isolates within each profile. The ten isolates without detected amino acid alterations in the targeted coding regions are presented separately in Supplementary Table 2. (**B**) Relative expression of *pmrA*, *pmrB*, *pmrD*, *pmrE*, *pmrK*, and *phoQ* compared with *K. pneumoniae* ATCC 700603. Colored dots beside isolate IDs indicate the corresponding lipid A profile shown in panel A. Blue shading indicates relative expression <1, white indicates values similar to the reference (1.0), and red shading indicates relative expression >1; NA, not available. qRT-PCR values represent descriptive relative expression measurements obtained from technical duplicates of the same RNA preparation. Acidic pH corresponds to pH 5.8. Short-term survival was assessed following a 1-h polymyxin B challenge at 10× the previously determined MIC for each isolate. “Detected” indicates recovery of viable cells following the polymyxin B challenge under the acidic condition, “Not detected” indicates no recovery of viable cells under the experimental conditions, and NA indicates not assessed

All isolates were resistant to polymyxin B, with MICs ranging from 3 to ≥ 64 µg/mL, and no mcr-1 to mcr-5 genes were detected. Genetic alterations in at least one of the investigated chromosomal loci were identified in 40 of the 50 isolates (80%), including nonsynonymous substitutions, premature stop codons, and/or deletions or insertions (Fig. 1A). Deletions involving *mgrB* were detected in *K. pneumoniae* isolates 110/17, 172/17, 149/21, 216/22, 292/22, 192/22, 209/22, and 281/22, whereas *mgrB* insertions were observed in isolates 1013/16, 58/16, 182/16, 346/16, 429/16, and 184/18. Deletions involving *crrB* were detected in isolates 216/22, 983/16, 641/21, and 230/22. Amino acid alterations were identified in *PmrA* in six isolates (E57G), *PmrB* in ten isolates (S180P, A223T, G233R, and V329L), *PhoQ* in ten isolates (V15F, V24G, E154*, K186R, V383L, and G479R), *MgrB* in sixteen isolates (L4*, L18*, Q22L, C27F, Q30*, C39Y, K43*, F44C, and P46S), and CrrB in three isolates (I27V, N2K, and Q296L). No amino acid alterations were detected in *PhoP*. The corresponding nucleotide substitutions are detailed in Supplementary Table 1, and amino acid sequence comparisons are provided in Supplementary Table 3.

### Lipid A remodeling in isolates without detected amino acid alterations in targeted resistance-associated loci

Ten isolates showed no amino acid alterations in the sequenced coding regions of *pmrAB*,* phoPQ*,* mgrB*, and *crrB*, despite exhibiting polymyxin B resistance and lipid A modifications detected by MALDI-TOF MS (Supplementary Table 2). For example, isolate Kp279/17 exhibited PEtN incorporation despite the absence of detected amino acid alterations in these loci and of *mcr*-like genes.

### Lipid A analysis

MALDI-TOF-MS analysis of lipid A revealed ions corresponding to hexa-acylated, biphosphorylated lipid A, with or without LpxO-associated hydroxylation. Additional ions at *m/z* 1921, 1972, 2079, and 2210 were consistent with structural modifications involving phosphate addition (MW, 80), L-Ara4N incorporation (MW, 131), and palmitoylation/C16 addition (MW, 238). Based on the combinations of these lipid A-associated ions, the 40 isolates with detected genetic alterations were distributed across five lipid A profiles, with two profiles predominating (*n* = 11 and *n* = 25), whereas the remaining three profiles comprised one, one, and two isolates, respectively (Fig. 1A).

### *In vivo* virulence assay

Based on the *G. mellonella* experiment, all fifty polymyxin-resistant, mcr-negative *K. pneumoniae* isolates were descriptively categorized into three groups (A–C): Group A, low/non-virulent phenotype; Group B, moderate-virulence phenotype; and Group C, high-virulence phenotype. These classifications refer specifically to relative larval mortality under the experimental conditions used in this study (Supplementary Fig. 1).

### Short-term survival following polymyxin B exposure under different pH conditions

Eighteen *K. pneumoniae* isolates carrying amino acid changes in *PmrA*, *PmrB*, or *PhoQ* were evaluated for short-term survival following polymyxin B exposure at pH 5.8 and 7.7. Viable cells were recovered after the 1-h polymyxin B challenge at 10× the previously determined MIC under the acidic condition (pH 5.8) for four isolates (Kp388/16, Kp1013/16, Kp100/17, and Kp322/17) (Fig. 1B and Supplementary Fig. 2).

### qRT-PCR expression analysis

Relative expression values varied across genes and isolates. Most measured values were at or below those observed for *K. pneumoniae* ATCC 700603. The highest relative expression values were observed for *pmrD* in isolate 58/16 (3.71-fold) and *pmrE* in isolate 172/17 (3.20-fold). Other comparatively high values included *pmrE* in isolates 10/21 (2.17-fold) and 288/17 (1.87-fold), *pmrK* in isolate 47/22 (2.00-fold), and *pmrD* in isolate 339/22 (1.84-fold) (Fig. 1B).

### Lipid A and WGS analysis of *E. coli* D12

MALDI-TOF-MS analysis of lipid A from the *E. coli* D12 mutant revealed modifications consistent with polymyxin resistance, including Ara4N, PEtN, and palmitoylation (Supplementary Fig. 3). The baseline *m/z* 1797 peak corresponded to biphosphorylated, hexa-acylated lipid A, while additional peaks indicated PEtN (MW, 123), Ara4N (MW, 131), palmitate (MW, 238), and combined additions (*m/z* 2159). QUAST analysis of the *E. coli* D12 assembly, considering contigs ≥ 500 bp, yielded 148 contigs with a total length of 5.15 Mb, a GC content of 50.29%, an N50 of 173,674 bp, an L50 of 10, and a largest contig of 458,325 bp. Based on the total number of sequenced bases relative to the assembly size, the estimated sequencing depth was approximately 155×. CheckM estimated genome completeness of 99.97% and contamination of 4.07%. In silico analyses identified non-synonymous substitutions in *PmrA* (S29G, I128N, G144S) and *PmrB* (H2R, E166A, D282G) (Supplementary Fig. 4).

## Discussion

Polymyxin resistance in *Klebsiella pneumoniae* has emerged as a multifactorial phenomenon driven by the interplay of genetic alterations, regulatory rewiring, and adaptive responses to environmental pressures. In this study, we combined antimicrobial susceptibility testing, targeted molecular analysis of canonical resistance loci, lipid A structural profiling, and phenotypic assays to characterize polymyxin-resistant KPC-producing *K. pneumoniae* isolates from Brazilian hospitals. This integrated approach allowed us to assess not only the prevalence of well-established resistance determinants but also the extent of phenotypic and adaptive heterogeneity within this clinically relevant population.

In parallel with resistance to polymyxins, a high frequency of resistance to ceftazidime–avibactam (CAZ-AVI) was observed among the studied isolates. CAZ-AVI is the first-line cephalosporin/β-lactamase inhibitor combination for treating infections caused by KPC-producing pathogens (Shields et al. 2015). While it is active against strains producing multiple β-lactamases, including KPC, resistance has rapidly emerged since its clinical introduction. Understanding the mechanisms underlying this resistance, such as β-lactamase variants, enzyme hyperexpression, reduced permeability, and efflux pump overexpression, is essential for predicting resistance patterns and guiding antimicrobial stewardship (Xu et al. 2022). In the present collection, sequencing of *bla*_KPC_ showed that all 42 isolates for which an allele could be unambiguously assigned carried *bla*_KPC−2_, despite the high frequency of CAZ-AVI resistance observed. Importantly, the presence of *bla*_KPC−2_ was not restricted to either resistant or susceptible isolates, indicating that KPC subtype alone does not explain the CAZ-AVI phenotype. Although KPC variants are well-established contributors to CAZ-AVI resistance, additional mechanisms, including β-lactamase hyperexpression, altered outer membrane permeability, porin defects, and efflux activity, may contribute to reduced susceptibility in KPC-producing *K. pneumoniae* (Shields et al. 2015; Xie et al. 2025; Lu et al. 2026). These mechanisms were not specifically investigated in the present study and therefore warrant further investigation. The correlation between phenotypic and genotypic profiles was largely consistent; however, isolate Kp 47/22 remained phenotypically susceptible to imipenem and meropenem despite carrying the *bla*_KPC_ gene. This observation reinforces that the presence of a resistance gene does not necessarily translate into phenotypic resistance, as gene expression may be impaired by insertions, deletions, or regulatory disruptions affecting gene activation, as previously reported (Salimnia et al. 2020; Ballaben et al. 2022).

Beyond resistance to β-lactam/β-lactamase inhibitor combinations, polymyxin resistance in this collection was further explored through targeted analysis of canonical chromosomal regulatory pathways. All isolates were resistant to polymyxin B, and no *mcr-1* to *mcr-5* genes were detected. Alterations were frequently identified in components of the PmrAB and PhoPQ two-component systems and in *mgrB*, whereas alterations affecting CrrB were less frequent. These pathways are well established in the regulation of lipid A remodeling and polymyxin susceptibility in *K. pneumoniae*. Nevertheless, because the clinical isolates were investigated by targeted sequencing rather than whole-genome sequencing, the contribution of additional chromosomal determinants cannot be excluded. The high proportion of isolates presenting amino acid substitutions, deletions, or insertions in these loci underscores the genetic diversity of canonical polymyxin resistance-associated pathways within this population, although the individual contribution of these alterations to the resistance phenotype cannot be determined from the present data without functional validation.

To contextualize the potential relevance of the detected alterations, individual variants were interpreted in light of their reported frequency, affected functional regions, prior associations with polymyxin resistance, and available functional evidence.

The *PmrA* E57G substitution, located in the phosphate receptor domain, has been repeatedly associated with polymyxin resistance (Aires et al. 2016; Binsker et al. 2022; Kao et al. 2024). Four amino acid substitutions were identified in *PmrB* using *K. pneumoniae* HS11286 as the reference: S180P, A223T, G233R, and V329L. A223T and G233R have previously been reported in polymyxin-resistant *K. pneumoniae* using the same HS11286 reference sequence employed in the present study (Mills et al. 2021; Chen et al. 2022), although their individual contribution to resistance has not been functionally established. Notably, sequence alignment of the *PmrB* proteins from HS11286 and MGH78578 showed that S180 in HS11286 corresponds to S203 in MGH78578 because of the difference in N-terminal length between the two reference proteins. Thus, the S180P substitution detected here corresponds to the S203P substitution recently investigated by Xiao et al. (2026). In that study, CRISPR-Cas9-based functional validation supported a causal contribution of S203P to colistin resistance, with reversion of the mutation restoring colistin susceptibility and suppressing lipid A modification pathways. Nevertheless, S180P occurred together with G233R in the isolate examined here, preventing attribution of its polymyxin resistance phenotype specifically to S180P. The functional significance of V329L remains unclear.

*PhoQ* alterations identified in this collection included V15F, V24G, E154*, K186R, V383L, and G479R. Among these, V24G has previously been identified in colistin-resistant *K. pneumoniae* and has functional support for involvement in resistance, as complementation with the corresponding wild-type gene restored colistin susceptibility (Sisti et al. 2022). V24G was also identified among colistin-resistant subpopulations derived from clinical isolates and predicted to have a deleterious effect on *PhoQ* (Sánchez-León et al. 2023). In contrast, the functional relevance of V15F, K186R, V383L, and G479R remains unclear. K186R and V383L represent conservative amino acid substitutions, whereas E154* introduces a premature stop codon and is therefore expected to result in a truncated *PhoQ* protein. However, the phenotypic consequences of these alterations were not experimentally determined in the present study. Because no knock-in, complementation, or isogenic mutant experiments were performed for the clinical isolates, the detected substitutions should be regarded as candidate resistance-associated variants unless supported by independent functional evidence. Importantly, functional evidence reported for individual substitutions in other strain backgrounds does not establish their causal contribution to polymyxin resistance in the isolates investigated here.

The predominant mechanism of polymyxin resistance in *K. pneumoniae* remains the loss of *MgrB* activity, which results in constitutive activation of the PhoPQ system (Khoshbayan et al. 2024). In this collection, multiple *mgrB* alterations were detected, including deletions, insertions, premature stop codons, and amino acid substitutions, several of which have been previously linked to polymyxin resistance (Vendrik et al. 2022; Yap et al. 2022; Camargo et al. 2023; Zahedi Bialvaei et al. 2023). While some substitutions, such as Q22L and C39Y, have been associated with resistance (Vendrik et al. 2022; Yap et al. 2022), others, including F44C, have been identified in both resistant and susceptible strains (Camargo et al. 2023), indicating that individual substitutions may not be sufficient to confer resistance on their own. Premature stop codons identified in our collection, including L4*, L18*, Q30*, and K43*, are expected to result in truncated *MgrB* proteins. Of note, Q30* has previously been observed during the *in vivo* emergence of colistin resistance in K. pneumoniae (Kong et al. 2021), and disruptive *mgrB* alterations were restricted to colistin-resistant isolates in a recent clinical collection (Xiao et al. 2026). Collectively, these observations are consistent with the established role of *mgrB* disruption in polymyxin resistance. However, the contribution of individual *mgrB* substitutions identified in this collection cannot be inferred from sequence association alone and requires functional validation.

Alterations involving the *crrAB* system were less frequent. This operon encodes the regulatory protein CrrA and the sensor kinase CrrB, which positively regulate *pmrC* expression via PmrAB (Poirel et al. 2017). Following sequence reanalysis using *K. pneumoniae* HS11286 as the reference, nonsynonymous CrrB substitutions were identified in only two isolates: I27V in Kp 1013/16 and N2K together with Q296L in Kp 47/22. I27V has been reported previously in colistin-resistant *K. pneumoniae* (Pishnian et al. 2019); however, more recent data showed this variant exclusively among colistin-susceptible isolates, indicating that I27V alone is unlikely to constitute a sufficient resistance determinant (Xiao et al. 2026). Q296L has also been described among polymyxin-resistant clinical *K. pneumoniae* isolates (Cadena et al. 2021), although its independent contribution to the resistance phenotype remains unresolved. The functional significance of N2K is likewise unknown. In addition, *crrB* deletions were detected in a subset of isolates. Taken together, these findings support a cautious interpretation of the CrrB variants identified here, as their presence alone does not establish a causal role in polymyxin resistance.

Notably, the relationship between the genetic alterations investigated and phenotypic resistance was not always straightforward in this collection. Several isolates showed no detectable amino acid alterations or disruptive events within the targeted coding regions of *pmrAB*,* phoPQ*,* mgrB*, and *crrB*, yet exhibited polymyxin resistance and lipid A modifications detected by MALDI-TOF MS. However, whole-genome sequencing and long-range PCR or other extended analyses specifically targeting regulatory regions were not performed for these clinical isolates. Therefore, alterations outside the sequenced coding regions, including promoter mutations, insertion sequences affecting *mgrB*, or regulatory changes involving *pmrD* or the *arn* operon, cannot be excluded. This finding highlights the complexity of polymyxin resistance expression and suggests the involvement of additional regulatory layers or compensatory mechanisms beyond the canonical loci examined. Gram-negative bacteria employ multiple strategies to resist polymyxins, including lipid A modification, efflux activity, capsule remodeling, and outer membrane protein regulation (Olaitan et al. 2014).

Although efflux pumps such as AcrAB and KpnEF have been implicated in colistin resistance (Padilla et al. 2010; Srinivasan and Rajamohan 2013), and efflux inhibition has reversed resistance in selected isolates (Sun et al. 2020; Pu et al. 2023), the overall contribution of proton motive force–dependent efflux remains uncertain (Ding et al. 2023), underscoring the need for further investigation. Future studies integrating whole-genome sequencing, regulatory-region analysis, transcriptomic profiling, and functional assays will be important to clarify the contribution of these alternative mechanisms to polymyxin resistance in isolates lacking detectable alterations in the targeted canonical loci.

Transcriptional analyses further illustrate the complexity of resistance regulation. Relative expression values varied across genes and isolates, with most measured values at or below those observed for the reference strain. Comparatively higher relative expression values were observed for *pmrD* and *pmrE* in a subset of isolates. Importantly, amino acid substitutions do not necessarily affect transcriptional activity, as mutations within coding regions typically influence protein function or stability rather than gene expression. In contrast, regulatory mutations affecting promoters or upstream elements are more directly associated with transcriptional changes (Kimball and Jefferson 2004). The variability in relative expression observed among isolates may therefore reflect the nature or position of the mutations, as well as compensatory regulatory mechanisms operating within these networks.

Lipid A structural analysis provided further insight into polymyxin resistance mechanisms. MALDI-TOF MS has emerged as a valuable tool for detecting lipid A modifications associated with polymyxin resistance in Gram-negative pathogens (Sorensen et al. 2020). The detection of modifications such as phosphoethanolamine and Ara4N addition, palmitoylation, and hydroxylation in the studied isolates aligns with previous reports describing reduced membrane electronegativity and decreased polymyxin binding (Liu et al. 2017; Doi and van Duin 2020; Solntceva et al. 2021). As a qualitative method, MALDI-TOF MS does not quantify the extent of these modifications, which may partly explain the observed heterogeneity in phenotypic resistance among isolates with similar lipid A profiles.

Collectively, the genetic, transcriptional, and lipid A structural data are consistent with a multifactorial basis for polymyxin resistance in *K. pneumoniae*, rather than supporting single deterministic genetic events. This complexity is further reflected in the lack of a direct association between resistance genotypes and virulence phenotypes observed in the *Galleria mellonella* infection model. Although this model provides a sensitive and cost-effective approach for virulence assessment, it does not fully recapitulate mammalian immunity, and caution is warranted when extrapolating these findings to human infection contexts. The absence of genotypic screening for virulence-associated loci in this study also limits genotype–phenotype correlation analyses and highlights an avenue for future research.

Environmental modulation experiments further underscored the heterogeneous response of *K. pneumoniae* to polymyxin exposure. Following the 1-h polymyxin B challenge at 10× the previously determined MIC, viable cells were recovered under the acidic condition (pH 5.8) for only a subset of isolates. These findings indicate heterogeneity in short-term survival under polymyxin stress across the tested environmental conditions and may reflect differences in the underlying genetic background. Environmental factors such as pH, divalent cation availability, temperature, and antimicrobial peptides are known to influence lipid A remodeling and outer membrane stability through two-component regulatory systems, including PmrAB and PhoPQ (Mitchell and Silhavy 2019; Simpson and Trent 2019; Huang et al. 2020). Importantly, the present assay evaluated short-term survival after polymyxin B exposure and was not designed to determine pH-dependent changes in MIC. The heterogeneous responses observed here mirror previous reports of variable adaptation and virulence among clinical *K. pneumoniae* isolates (Insua et al. 2013; Bruchmann et al. 2021).

Finally, analysis of the laboratory-evolved *Escherichia coli* D12 mutant under polymyxin selective pressure revealed lipid A modifications and amino acid substitutions in *PmrA* and *PmrB* consistent with previous reports in polymyxin-resistant *E. coli* (Sato et al. 2018). Although the contribution of the novel *PmrB* D282G substitution remains unclear, these findings illustrate convergent adaptive strategies across *Enterobacterales* and support the broader relevance of regulatory network remodeling in polymyxin resistance.

Taken together, these observations underscore that polymyxin resistance in KPC-producing *K. pneumoniae* reflects a dynamic and multifactorial adaptive process shaped by genetic alterations, regulatory interactions, lipid A remodeling, and environmental context. By integrating molecular, structural, and phenotypic data, this study contributes to a more nuanced understanding of resistance evolution in a high-risk clinical pathogen and highlights the importance of integrated surveillance approaches for interpreting antimicrobial resistance mechanisms in microbiology reference laboratories.

### Study limitations

Several limitations should be considered when interpreting the present findings. First, the clinical *K. pneumoniae* isolates were investigated using targeted PCR and Sanger sequencing rather than whole-genome sequencing. Consequently, additional resistance determinants, alterations in regulatory regions, insertion sequences affecting *mgrB*, and changes involving other loci such as *pmrD* or the *arn* operon cannot be excluded. The absence of molecular typing or genome-wide phylogenetic analysis also prevents assessment of clonal relatedness among the isolates; therefore, recurrent variants may reflect shared lineage backgrounds or clonal dissemination rather than independent mutational events. Second, no knock-in, complementation, or isogenic mutant experiments were performed for clinical isolates, limiting causal inference regarding the contribution of individual amino acid substitutions to polymyxin resistance. Third, qRT-PCR measurements were obtained from a single RNA preparation per isolate and analyzed in technical duplicate. Consequently, biological variability could not be assessed, and inferential statistical analyses were not performed; the transcriptional findings were therefore interpreted descriptively. In addition, virulence was evaluated using the *Galleria mellonella* model, which does not fully reproduce mammalian host immunity, and virulence-associated genes were not systematically investigated. Finally, the pH experiment assessed short-term bacterial survival following polymyxin B exposure and was not designed to determine pH-dependent changes in MIC. Future studies integrating whole-genome sequencing, clonal analysis, regulatory-region characterization, transcriptomic approaches, and functional validation will be important to further define the mechanisms underlying polymyxin resistance in this collection.

## Conclusions

This study demonstrates a high burden of antimicrobial resistance among polymyxin-resistant Klebsiella pneumoniae isolates recovered from Brazilian hospitals, with all isolates carrying *bla*_KPC_ and more than half co-harboring *bla*_CTX−M−type_ genes. The high frequency of ceftazidime–avibactam resistance further highlights the clinical relevance of this collection and reinforces the need for continuous antimicrobial resistance surveillance.

Through an integrated microbiological approach, we identified diverse alterations in canonical polymyxin resistance-associated pathways, particularly involving *mgrB*,* pmrAB*,* phoQ*, and *crrB*, together with lipid A remodeling detected by MALDI-TOF MS. However, the occurrence of individual amino acid substitutions should not be interpreted as evidence of causality, and several isolates exhibited polymyxin resistance despite the absence of detectable amino acid alterations in the targeted coding regions, supporting the multifactorial nature of this phenotype.

Phenotypic analyses further demonstrated substantial heterogeneity among isolates. No direct association between the investigated resistance-associated alterations and virulence phenotypes was observed in the *Galleria mellonella* model, while short-term survival following polymyxin B exposure at pH 5.8 and 7.7 differed in only a subset of isolates, indicating a strain-dependent response to environmental conditions. The laboratory-evolved *Escherichia coli* D12 mutant also exhibited lipid A remodeling and regulatory-system alterations following polymyxin selective pressure, further illustrating bacterial adaptation under antimicrobial exposure.

Overall, these findings support a model in which polymyxin resistance reflects the combined contribution of genetic variation, regulatory responses, lipid A remodeling, and environmental context rather than a single deterministic mechanism. Integrating targeted molecular analysis with structural and phenotypic approaches may therefore improve the interpretation and surveillance of polymyxin resistance in clinically relevant *K. pneumoniae* populations.

## Supplementary information

Below is the link to the electronic supplementary material.

Supplementary File 1 (DOCX 24.9 KB)

Supplementary File 2 (DOCX 17.9 KB)

Supplementary File 3 (DOCX 23.5 KB)

Supplementary File 4 (DOCX 245 KB)

Supplementary File 5 (DOCX 395 KB)

Supplementary File 6 (DOCX 191 KB)

Supplementary File 7 (DOCX 646 KB)

## Data Availability

The sequencing data for *E. coli* D12 have been deposited in the NCBI under BioProject accession [PRJNA1248546], BioSample accession [SAMN62523585], SRA accession [SRR40268814].
