# Supplementary material for: An integrated molecular, phenotypic, and adaptive analysis of polymyxin resistance in KPC-producing Klebsiella pneumoniae
Source: World J Microbiol Biotechnol. 2026 Nov 11;42(11):652. doi: 10.1007/s11274-026-05240-1 (PMC13754935; doi:10.1007/s11274-026-05240-1)
Supplement: Supplementary file 1 [file 11274_2026_5240_MOESM1_ESM.docx]

Supplementary Table 1 Nucleotide changes and corresponding amino acid alterations in targeted polymyxin resistance-associated loci among clinical *Klebsiella pneumoniae* isolates.

| Isolate/Year | Nucleotides → Protein | | | | | |
| --- | --- | --- | --- | --- | --- | --- |
|  | ***pmrA*** | ***pmrB*** | ***phoP*** | ***phoQ*** | ***mgrB*** | ***crrB*** |
| 388/16 | A170G, G480A → **E57G** | C250T, C330G, C433G, C697T → **G233R** | WT → WT | C219T, A309C, C1005T → WT | G116A → **C39Y** | WT → WT |
| 1013/16 | T132C, G282A, C294T, C472T, G40A, G504A, G552A → WT | C250T, C330G, C433G, A790G, T802A, T1035C → **A223T, G233R** | T404C, T705C → WT | WT → WT | Insertion | A202G, A207C, A1184G → **I27V** |
| 100/17 | A170G, G480A → **E57G** | C250T, C330G, C433G, C697T → **G233R** | WT → WT | WT → WT | G116A → **C39Y** | C118T, A207C, C1199G → WT |
| 322/17 | A170G, G480A → **E57G** | C250T, C330G, C433G, C697T → **G233R** | WT → WT | C219T, C564T, C1023T → WT | WT → WT | C118T, A207C → WT |
| 58/16 | WT → WT | WT → WT | WT → WT | A557G → **K186R** | Insertion | WT → WT |
| 182/16 | WT → WT | C18G, A237T, T279C, G606C, G679A, G709C, G789A, C809A → **A223T, G233R** | T404C, T705C → WT | C219T, A309C, C1005T → WT | Insertion | WT → WT |
| 346/16 | WT → WT | WT → WT | WT → WT | WT → WT | Insertion | WT → WT |
| 790/16 | A170G, G480A → **E57G** | C250T, C330G, C433G, A790G, T1035C → **S180P**, **G233R** | WT → WT | C219T, C1023T, G1047T → **V383L** | A65T → **Q22L** | C118T, A207C, C1199G → WT |
| 110/17 | A170G, G480A → **E57G** | C250T, C330G, C433G, C697T → **G233R** | WT → WT | C219T, G460T, C564T, C623G, C1023T → **E154*** | Deletion | C118T, A207C, C1199G → WT |
| 172/17 | WT → WT | C42G, T1035C → **V329L** | WT → WT | WT → WT | Deletion | A207C, A1010T → WT |
| 288/17 | WT → WT | C250T, C330G, C433G, C697T → **G233R** | WT → WT | WT → WT | C88T → **Q30*** | A207C → WT |
| 10/21 | WT → WT | C24G → WT | WT → WT | T71G → **V24G** | WT → WT | A207C, A1010T → WT |
| 48/21 | WT → WT | A184C → WT | WT → WT | T71G → **V24G** | WT → WT | A207C, A1010T → WT |
| 640/21 | WT → WT | WT → WT | WT → WT | T71G → **V24G** | WT → WT | A207C, A1010T → WT |
| 47/22 | WT → WT | WT → WT | WT → WT | T71G → **V24G** | WT → WT | C129A, A1010T → **N2K, Q296L** |
| 216/22 | WT → WT | C250T, C330G, C433G, A790G, T802A, T1035C → **A223T, G233R** | WT → WT | WT → WT | Deletion | Deletion |
| 256/22 | WT → WT | WT → WT | WT → WT | T71G, G1435C → **V24G, G479R** | WT → WT | WT → WT |
| 259/22 | A170G, G480A → **E57G** | WT → WT | WT → WT | WT → WT | C136T → **P46S** | C118T, A207C, C1199G → WT |
| 292/22 | WT → WT | WT → WT | WT → WT | G43T → **V15F** | Deletion | WT → WT |
| 339/22 | WT → WT | WT → WT | WT → WT | T71G → **V24G** | WT → WT | WT → WT |
| 429/16 | WT → WT | WT → WT | WT → WT | WT → WT | Insertion | WT → WT |
| 184/18 | WT → WT | WT → WT | WT → WT | WT → WT | Insertion | WT → WT |
| 149/21 | WT → WT | WT → WT | WT → WT | WT → WT | Deletion | WT → WT |
| 192/22 | WT → WT | WT → WT | WT → WT | WT → WT | Deletion | WT → WT |
| 209/22 | WT → WT | WT → WT | WT → WT | WT → WT | Deletion | WT → WT |
| 281/22 | WT → WT | WT → WT | WT → WT | WT → WT | Deletion | WT → WT |
| 247/21 | WT → WT | WT → WT | WT → WT | WT → WT | T131G → **F44C** | WT → WT |
| 276/21 | WT → WT | WT → WT | WT → WT | WT → WT | T131G → **F44C** | WT → WT |
| 426/21 | WT → WT | WT → WT | WT → WT | WT → WT | T131G → **F44C** | A207C, A1010T → WT |
| 185/21 | WT → WT | WT → WT | WT → WT | WT → WT | G82T → **C27F** | WT → WT |
| 335/21 | WT → WT | WT → WT | WT → WT | WT → WT | T11G → **L4*** | A207C, A1010T → WT |
| 593/21 | WT → WT | WT → WT | WT → WT | WT → WT | T11G → **L4*** | A207C, A1010T → WT |
| 114/21 | WT → WT | WT → WT | WT → WT | WT → WT | T11G → **L4*** | A207C, A1010T → WT |
| 18/22 | WT → WT | WT → WT | WT → WT | WT → WT | T53A → **L18*** | A207C, A1010T → WT |
| 38/22 | WT → WT | WT → WT | WT → WT | WT → WT | T53A → **L18*** | WT → WT |
| 48/22 | WT → WT | WT → WT | WT → WT | WT → WT | T53A → **L18*** | A207C, A1010T → WT |
| 480/17 | WT → WT | WT → WT | WT → WT | WT → WT | T127A → **K43*** | WT → WT |
| 983/16 | WT → WT | WT → WT | WT → WT | WT → WT | WT → WT | Deletion |
| 641/21 | WT → WT | WT → WT | WT → WT | WT → WT | WT → WT | Deletion |
| 230/22 | WT → WT | WT → WT | WT → WT | WT → WT | WT → WT | Deletion |
| 237/16 | WT → WT | WT → WT | WT → WT | T71G, A208G, G386A, A710G, T848C → WT | WT → WT | WT → WT |
| 909/16 | WT → WT | WT → WT | WT → WT | WT → WT | WT → WT | WT → WT |
| 279/17 | WT → WT | WT → WT | WT → WT | WT → WT | WT → WT | WT → WT |
| 27/21 | WT → WT | WT → WT | WT → WT | A309C, C1185T, C1194T → WT | WT → WT | C1203T → WT |
| 72/21 | WT → WT | WT → WT | WT → WT | WT → WT | WT → WT | WT → WT |
| 11/22 | WT → WT | WT → WT | WT → WT | WT → WT | WT → WT | A207C, A1010T → WT |
| 34/22 | WT → WT | WT → WT | WT → WT | WT → WT | WT → WT | WT → WT |
| 36/22 | WT → WT | WT → WT | WT → WT | WT → WT | WT → WT | A207C, A1010T → WT |
| 288/22 | WT → WT | WT → WT | WT → WT | A309C, C1185T, C1194T → WT | WT → WT | WT → WT |
| 324/22 | WT → WT | WT → WT | WT → WT | WT → WT | WT → WT | A207C, A1010T → WT |

Amino acid sequences were deduced from Sanger-sequenced loci and compared with the corresponding sequences from *Klebsiella pneumoniae* subsp. *pneumoniae* HS11286 (GenBank accession CP003200.1). WT → WT indicates no nucleotide or amino acid difference from the reference sequence. Nucleotide substitutions followed by → WT indicate synonymous substitutions without alteration of the deduced amino acid sequence. The asterisk (*) indicates a premature stop codon.
