# Supplementary material for: An integrated molecular, phenotypic, and adaptive analysis of polymyxin resistance in KPC-producing Klebsiella pneumoniae
Source: World J Microbiol Biotechnol. 2026 Nov 11;42(11):652. doi: 10.1007/s11274-026-05240-1 (PMC13754935; doi:10.1007/s11274-026-05240-1)
Supplement: Supplementary file 2 [file 11274_2026_5240_MOESM2_ESM.docx]

Supplementary Table 2 Polymyxin B MICs and lipid A profiles of *Klebsiella pneumoniae* isolates without detected amino acid alterations in targeted polymyxin resistance-associated loci.

| **Isolate/Year** | **Targeted chromosomal loci ^a^** | | | | | **MS-Lipid A^b^** | **MIC PMB (µg/mL)^c^** |
| --- | --- | --- | --- | --- | --- | --- | --- |
|  | ***pmrA*** | ***pmrB*** | ***phoQ*** | ***mgrB*** | ***crrB*** |  |  |
| 237/16 | NAA | NAA | NAA | NAA | NAA | LpxO + LpxO + L-Ara4N + C16 | 48 |
| 909/16 | NAA | NAA | NAA | NAA | NAA | LpxO + L-Ara4N + C16 | 16 |
| 279/17 | NAA | NAA | NAA | NAA | NAA | PEtN + C16 | 3 |
| 27/21 | NAA | NAA | NAA | NAA | NAA | Phosphate + L-Ara4N + C16 + L-Ara4N + C16 | 16 |
| 72/21 | NAA | NAA | NAA | NAA | NAA |  | 32 |
| 11/22 | NAA | NAA | NAA | NAA | NAA |  | 64 |
| 34/22 | NAA | NAA | NAA | NAA | NAA |  | 64 |
| 36/22 | NAA | NAA | NAA | NAA | NAA |  | 64 |
| 288/22 | NAA | NAA | NAA | NAA | NAA |  | 4 |
| 324/22 | NAA | NAA | NAA | NAA | NAA |  | 64 |

^a^ NAA, no amino acid alteration detected in the deduced protein sequence obtained from the targeted Sanger-sequenced coding region compared with the corresponding sequence of Klebsiella pneumoniae subsp. pneumoniae HS11286 (GenBank accession CP003200.1). This designation does not exclude alterations in regulatory regions, insertion sequences, or other genomic loci not investigated in this study.

^b^ Lipid A characterization was performed using matrix-assisted laser desorption/ionization time-of-flight mass spectrometry (MALDI-TOF MS) in negative-ion reflectron mode. Spectra were acquired and processed using flexControl and flexAnalysis software, version 3.4 (Bruker Daltonics Inc.). L-Ara4N: 4-amino-4-deoxy-L-arabinose (aminoarabinose); PEtN: phosphoethanolamine; C-16: palmitate. Addition or removal of a hydroxyl group in an acyl chain, mediated by LpxO.

^c^ PMB: polymyxin B; according to BrCAST , the recommended breakpoint values are: ≤2 µg/mL (Susceptible), >2 µg/mL (Resistant).
