# Supplementary material for: An integrated molecular, phenotypic, and adaptive analysis of polymyxin resistance in KPC-producing Klebsiella pneumoniae
Source: World J Microbiol Biotechnol. 2026 Nov 11;42(11):652. doi: 10.1007/s11274-026-05240-1 (PMC13754935; doi:10.1007/s11274-026-05240-1)
Supplement: Supplementary file 3 [file 11274_2026_5240_MOESM3_ESM.docx]

Supplementary Table 3 - Amino acid sequence comparison of polymyxin resistance-associated proteins in clinical *Klebsiella pneumoniae* isolates.

| Protein / isolate ID | Amino acid sequence | Amino acid alteration |
| --- | --- | --- |
| **PmrA** | MKI LVI EDD ALL LQG LIL AMQ SEG YVC DGV STA HEA ALS LAS NHY SLI VLD LGL PDE DGL HFL SEM RRE KMT QPV LIL TAR DTL EDR ISG LDT GAD DYL VKP FAL EEL NAR IRA LLR RHN NQG DNE ISV GNL RLN VTR RLV WLG ETA LDL TPK EYA LLS RLM MKA GSP VHR EIL YND IYS WDN EPA TNT LEV HIH NLR EKI GKS RIR TVR GFG YML ANN IDT E* | WT |
|  |  |  |
| 388/16 | MKI LVI EDD ALL LQG LIL AMQ SEG YVC DGV STA HEA ALS LAS NHY SLI VLD LGL PDG DGL HFL SEM RRE KMT QPV LIL TAR DTL EDR ISG LDT GAD DYL VKP FAL EEL NAR IRA LLR RHN NQG DNE ISV GNL RLN VTR RLV WLG ETA LDL TPK EYA LLS RLM MKA GSP VHR EIL YND IYS WDN EPA TNT LEV HIH NLR EKI GKS RIR TVR GFG YML ANN IDT E* | E57G |
| 100/17 |  |  |
| 322/17 |  |  |
| 790/16 |  |  |
| 110/17 |  |  |
| 259/22 |  |  |
|  |  |  |
| **PmrB** | MVC QLI SVF WLW HES KEQ IQL LVA SAI EGH NNQ KHV EHE VRE AVA SLL VPS LLI VGL ALY ISM LAV RKI TRP LSR LQS ELE NRT PDN LTP IVL SES VPE VTA VTT ALN QLV SRL NLT LDR ERL FTA DVA HEL RTP LAG LRL HLE LLA KVH GMG VDP LIQ RLD QMT TSI SQL LQL ARV GQS FSA GSY QQV LLL DDV VKP LQD ELE AML AQR QQR LLL TDI ENE AVV SGD ATL IGV ILR NLV ENA HRY SPE GST IRV SVK AGL MPV MAV EDE GPG IDE AKS GEL SKA FVR MDS RYG GIG LGL SIV TRI AQL HDA QFF LHN RQP GPG VRA WVL FPQ RGG QNV STH | WT |
|  |  |  |
| 388/16 | MVC QLI SVF WLW HES KEQ IQL LVA SAI EGH NNQ KHV EHE VRE AVA SLL VPS LLI VGL ALY ISM LAV RKI TRP LSR LQS ELE NRT PDN LTP IVL SES VPE VTA VTT ALN QLV SRL NLT LDR ERL FTA DVA HEL RTP LAG LRL HLE LLA KVH GMG VDP LIQ RLD QMT TSI SQL LQL ARV GQS FSA GSY QQV LLL DDV VKP LQD ELE AML AQR QQR LLL TDI ENE AVV SGD ATL IRV ILR NLV ENA HRY SPE GST IRV SVK AGL MPV MAV EDE GPG IDE AKS GEL SKA FVR MDS RYG GIG LGL SIV TRI AQL HDA QFF LHN RQP GPG VRA WVL FPQ RGG QNV STH | G233R |
| 100/17 |  |  |
| 322/17 |  |  |
| 110/17 |  |  |
| 288/17 |  |  |
|  |  |  |
| 790/16 | MVC QLI SVF WLW HES KEQ IQL LVA SAI EGH NNQ KHV EHE VRE AVA SLL VPS LLI VGL ALY ISM LAV RKI TRP LSR LQS ELE NRT PDN LTP IVL SES VPE VTA VTT ALN QLV SRL NLT LDR ERL FTA DVA HEL RTP LAG LRL HLE LLA KVH GMG VDP LIQ RLD QMT TSI SQL LQL ARV GQP FSA GSY QQV LLL DDV VKP LQD ELE AML AQR QQR LLL TDI ENE AVV SGD ATL IRV ILR NLV ENA HRY SPE GST IRV SVK AGL MPV MAV EDE GPG IDE AKS GEL SKA FVR MDS RYG GIG LGL SIV TRI AQL HDA QFF LHN RQP GPG VRA WVL FPQ RGG QNV STH | S180P, G233R |
|  |  |  |
| 182/16  1013/16  216/22 | MVC QLI SVF WLW HES KEQ IQL LVA SAI EGH NNQ KHV EHE VRE AVA SLL VPS LLI VGL ALY ISM LAV RKI TRP LSR LQS ELE NRT PDN LTP IVL SES VPE VTA VTT ALN QLV SRL NLT LDR ERL FTA DVA HEL RTP LAG LRL HLE LLA KVH GMG VDP LIQ RLD QMT TSI SQL LQL ARV GQS FSA GSY QQV LLL DDV VKP LQD ELE AML AQR QQR LLL TDI ENE TVV SGD ATL IRV ILR NLV ENA HRY SPE GST IRV SVK AGL MPV MAV EDE GPG IDE AKS GEL SKA FVR MDS RYG GIG LGL SIV TRI AQL HDA QFF LHN RQP GPG VRA WVL FPQ RGG QNV STH | A223T, G233R |
|  |  |  |
| 172/17 | MVC QLI SVF WLW HES KEQ IQL LVA SAI EGH NNQ KHV EHE VRE AVA SLL VPS LLI VGL ALY ISM LAV RKI TRP LSR LQS ELE NRT PDN LTP IVL SES VPE VTA VTT ALN QLV SRL NLT LDR ERL FTA DVA HEL RTP LAG LRL HLE LLA KVH GMG VDP LIQ RLD QMT TSI SQL LQL ARV GQS FSA GSY QQV LLL DDV VKP LQD ELE AML AQR QQR LLL TDI ENE AVV SGD ATL IGV ILR NLV ENA HRY SPE GST IRV SVK AGL MPV MAV EDE GPG IDE AKS GEL SKA FVR MDS RYG GIG LGL SIV TRI AQL HDA QFF LHN RQP GPG VRA WLL FPQ RGG QNV STH | V329L |
|  |  |  |
| **PhoQ** | MKG LLR HIF PLS LRV RFL LAT AGV VLV LSL AYG MVA LVG YSV SFD KTT FRL LRG ESN LFY MLA RWE NGA IDV DIP ENL NME SPT VTL IYD EQG KLL WAQ RDV PWL AKR IQP EWL KRN GFH EIE ADV DSS SML LRN NHE IQE QLD AIR EQG DDS EMT HSV AIN LYP ATS KMP QLS IVV VDT IPV ELK RSY MVW SWF VYV LAA NLL LVI PLL WVA AWW SLR PIE SLA KEV REL EEH HRE KLN PNT TRE LTR LVS NLN RLV RSE RER YDK YRT TLT DLT HSL KTP LAV MQS TLR SLR GEK ISV DEA EPV MLE QIS RIS QQI GYY LHR ASM RSG GTL LSR ELH PIA PLL DSL TSA LNK VYQ RKG VNI SLD ISP EIT FVG EQN DFM EVM GNV LDN ACK YCL EFV EVS VRQ TTD SHL HIL VED DGP GIP QSQ RRA VFD RGQ RAD TLR PGQ GVG LSV ARE IVE QYD GEI IAG ESL LGG ACM EVV FGR QQM EDK QS* | WT |
|  |  |  |
| 10/21 | MKG LLR HIF PLS LRV RFL LAT AGG VLV LSL AYG MVA LVG YSV SFD KTT FRL LRG ESN LFY MLA RWE NGA IDV DIP ENL NME SPT VTL IYD EQG KLL WAQ RDV PWL AKR IQP EWL KRN GFH EIE ADV DSS SML LRN NHE IQE QLD AIR EQG DDS EMT HSV AIN LYP ATS KMP QLS IVV VDT IPV ELK RSY MVW SWF VYV LAA NLL LVI PLL WVA AWW SLR PIE SLA KEV REL EEH HRE KLN PNT TRE LTR LVS NLN RLV RSE RER YDK YRT TLT DLT HSL KTP LAV MQS TLR SLR GEK ISV DEA EPV MLE QIS RIS QQI GYY LHR ASM RSG GTL LSR ELH PIA PLL DSL TSA LNK VYQ RKG VNI SLD ISP EIT FVG EQN DFM EVM GNV LDN ACK YCL EFV EVS VRQ TTD SHL HIL VED DGP GIP QSQ RRA VFD RGQ RAD TLR PGQ GVG LSV ARE IVE QYD GEI IAG ESL LGG ACM EVV FGR QQM EDK QS* | V24G |
| 48/21 |  |  |
| 640/21 |  |  |
| 47/22 |  |  |
| 339/22 |  |  |
|  |  |  |
| 292/22 | MKG LLR HIF PLS LRF RFL LAT AGV VLV LSL AYG MVA LVG YSV SFD KTT FRL LRG ESN LFY MLA RWE NGA IDV DIP ENL NME SPT VTL IYD EQG KLL WAQ RDV PWL AKR IQP EWL KRN GFH EIE ADV DSS SML LRN NHE IQE QLD AIR EQG DDS EMT HSV AIN LYP ATS KMP QLS IVV VDT IPV ELK RSY MVW SWF VYV LAA NLL LVI PLL WVA AWW SLR PIE SLA KEV REL EEH HRE KLN PNT TRE LTR LVS NLN RLV RSE RER YDK YRT TLT DLT HSL KTP LAV MQS TLR SLR GEK ISV DEA EPV MLE QIS RIS QQI GYY LHR ASM RSG GTL LSR ELH PIA PLL DSL TSA LNK VYQ RKG VNI SLD ISP EIT FVG EQN DFM EVM GNV LDN ACK YCL EFV EVS VRQ TTD SHL HIL VED DGP GIP QSQ RRA VFD RGQ RAD TLR PGQ GVG LSV ARE IVE QYD GEI IAG ESL LGG ACM EVV FGR QQM EDK QS* | V15F |
|  |  |  |
| 58/16 | MKG LLR HIF PLS LRV RFL LAT AGV VLV LSL AYG MVA LVG YSV SFD KTT FRL LRG ESN LFY MLA RWE NGA IDV DIP ENL NME SPT VTL IYD EQG KLL WAQ RDV PWL AKR IQP EWL KRN GFH EIE ADV DSS SML LRN NHE IQE QLD AIR EQG DDS EMT HSV AIN LYP ATS KMP QLS IVV VDT IPV ELR RSY MVW SWF VYV LAA NLL LVI PLL WVA AWW SLR PIE SLA KEV REL EEH HRE KLN PNT TRE LTR LVS NLN RLV RSE RER YDK YRT TLT DLT HSL KTP LAV MQS TLR SLR GEK ISV DEA EPV MLE QIS RIS QQI GYY LHR ASM RSG GTL LSR ELH PIA PLL DSL TSA LNK VYQ RKG VNI SLD ISP EIT FVG EQN DFM EVM GNV LDN ACK YCL EFV EVS VRQ TTD SHL HIL VED DGP GIP QSQ RRA VFD RGQ RAD TLR PGQ GVG LSV ARE IVE QYD GEI IAG ESL LGG ACM EVV FGR QQM EDK QS* | K186R |
|  |  |  |
| 790/16 | MKG LLR HIF PLS LRV RFL LAT AGV VLV LSL AYG MVA LVG YSV SFD KTT FRL LRG ESN LFY MLA RWE NGA IDV DIP ENL NME SPT VTL IYD EQG KLL WAQ RDV PWL AKR IQP EWL KRN GFH EIE ADV DSS SML LRN NHE IQE QLD AIR EQG DDS EMT HSV AIN LYP ATS KMP QLS IVV VDT IPV ELK RSY MVW SWF VYV LAA NLL LVI PLL WVA AWW SLR PIE SLA KEV REL EEH HRE KLN PNT TRE LTR LVS NLN RLV RSE RER YDK YRT TLT DLT HSL KTP LAV MQS TLR SLR GEK ISV DEA EPV MLE QIS RIS QQI GYY LHR ASM RSG GTL LSR ELH PIA PLL DSL TSA LNK VYQ RKG VNI SLD ISP EIT FVG EQN DFM ELM GNV LDN ACK YCL EFV EVS VRQ TTD SHL HIL VED DGP GIP QSQ RRA VFD RGQ RAD TLR PGQ GVG LSV ARE IVE QYD GEI IAG ESL LGG ACM EVV FGR QQM EDK QS* | V383L |
|  |  |  |
| 110/17 | MKG LLR HIF PLS LRV RFL LAT AGV VLV LSL AYG MVA LVG YSV SFD KTT FRL LRG ESN LFY MLA RWE NGA IDV DIP ENL NME SPT VTL IYD EQG KLL WAQ RDV PWL AKR IQP EWL KRN GFH EIE ADV DSS SML LRN NHE IQE QLD AIR EQG DDS * | E154* |
|  |  |  |
| 256/22 | MKG LLR HIF PLS LRV RFL LAT AGG VLV LSL AYG MVA LVG YSV SFD KTT FRL LRG ESN LFY MLA RWE NGA IDV DIP ENL NME SPT VTL IYD EQG KLL WAQ RDV PWL AKR IQP EWL KRN GFH EIE ADV DSS SML LRN NHE IQE QLD AIR EQG DDS EMT HSV AIN LYP ATS KMP QLS IVV VDT IPV ELK RSY MVW SWF VYV LAA NLL LVI PLL WVA AWW SLR PIE SLA KEV REL EEH HRE KLN PNT TRE LTR LVS NLN RLV RSE RER YDK YRT TLT DLT HSL KTP LAV MQS TLR SLR GEK ISV DEA EPV MLE QIS RIS QQI GYY LHR ASM RSG GTL LSR ELH PIA PLL DSL TSA LNK VYQ RKG VNI SLD ISP EIT FVG EQN DFM EVM GNV LDN ACK YCL EFV EVS VRQ TTD SHL HIL VED DGP GIP QSQ RRA VFD RGQ RAD TLR PGQ GVG LSV ARE IVE QYD GEI IAG ESL LGG ACM EVV FRR QQM EDK QS* | G479R, V24G |
|  |  |  |
| **MgrB** | VKK LRW VLL IVI IAG CLL LWT QML NVM CDQ DVQ FFS GIC TIN KFI PW* | WT |
|  |  |  |
| 247/21 | VKK LRW VLL IVI IAG CLL LWT QML NVM CDQ DVQ FFS GIC TIN KCI PW* | F44C |
| 276/21 |  |  |
| 426/21 |  |  |
|  |  |  |
| 114/21 | VKK * | L4* |
| 335/21 |  |  |
| 593/21 |  |  |
|  |  |  |
| 18/22 | VKK LRW VLL IVI IAG CL* | L18* |
| 38/22 |  |  |
| 48/22 |  |  |
|  |  |  |
| 388/16 | VKK LRW VLL IVI IAG CLL LWT QML NVM CDQ DVQ FFS GIY TIN KFI PW* | C39Y |
| 100/17 |  |  |
|  |  |  |
| 790/16 | VKK LRW VLL IVI IAG CLL LWT LML NVM CDQ DVQ FFS GIC TIN KFI PW* | Q22L |
|  |  |  |
| 288/17 | VKK LRW VLL IVI IAG CLL LWT QML NVM CD* | Q30* |
|  |  |  |
| 480/17 | VKK LRW VLL IVI IAG CLL LWT QML NVM CDQ DVQ FFS GIC TIN * | K43* |
|  |  |  |
| 185/21 | VKK LRW VLL IVI IAG CLL LWT QML NVM FDQ DVQ FFS GIC TIN KFI PW* | C27F |
|  |  |  |
| 259/22 | VKK LRW VLL IVI IAG CLL LWT QML NVM CDQ DVQ FFS GIC TIN KFI SW* | P46S |
|  |  |  |
| **CrrB** | MNK ETI LSR QIL TYM LLL TFV IIA IAI LGS YLF YSF LIY YLP GGM NEG SED AMT FLD WMW ILM ASI TSL VVA LFF TVK LSA RIF NPL NDV AYS LKQ ISQ GNL SAR AFN SSS KLG EMN RLV NDF NEM AEK LQT LDA QRN LWN AAI AHE LRT PVT ILR GRL QGL VDG VFE PEP SLF RNL LKQ TEG LTN LIE DLR VVS SSG GAG YSL MLS EVD LKA TIS NAL DTF WPD FDK KHF KIV TEI NQQ YCV CDP LRI IQC LTV LFD NAL KYS TSQ TLL IKN GIT GND NFI IVQ DQG PGI PEE LQK SLF QPF QRG EYA KNI NPE GCG LGL SVV KAI MCA HGG DVS YSL TPE NGS LFK LSW PV* | WT |
|  |  |  |
| 1013/16 | MNK ETI LSR QIL TYM LLL TFV IIA IAV LGS YLF YSF LIY YLP GGM NEG SED AMT FLD WMW ILM ASI TSL VVA LFF TVK LSA RIF NPL NDV AYS LKQ ISQ GNL SAR AFN SSS KLG EMN RLV NDF NEM AEK LQT LDA QRN LWN AAI AHE LRT PVT ILR GRL QGL VDG VFE PEP SLF RNL LKQ TEG LTN LIE DLR VVS SSG GAG YSL MLS EVD LKA TIS NAL DTF WPD FDK KHF KIV TEI NQQ YCV CDP LRI IQC LTV LFD NAL KYS TSQ TLL IKN GIT GND NFI IVQ DQG PGI PEE LQK SLF QPF QRG EYA KNI NPE GCG LGL SVV KAI MCA HGG DVS YSL TPE NGS LFK LSW PV* | I27V |
|  |  |  |
| 47/22 | MKK ETI LSR QIL TYM LLL TFV IIA IAI LGS YLF YSF LIY YLP GGM NEG SED AMT FLD WMW ILM ASI TSL VVA LFF TVK LSA RIF NPL NDV AYS LKQ ISQ GNL SAR AFN SSS KLG EMN RLV NDF NEM AEK LQT LDA QRN LWN AAI AHE LRT PVT ILR GRL QGL VDG VFE PEP SLF RNL LKQ TEG LTN LIE DLR VVS SSG GAG YSL MLS EVD LKA TIS NAL DTF WPD FDK KHF KIV TEI NQQ YCV CDP LRI IQC LTV LFD NAL KYS TSQ TLL IKN GIT GND NFI IVQ DQG PGI PEE LLK SLF QPF QRG EYA KNI NPE GCG LGL SVV KAI MCA HGG DVS YSL TPE NGS LFK LSW PV* | N2K, Q296L |

Amino acid sequences obtained from Sanger-sequenced loci were compared with the corresponding protein sequences from *Klebsiella pneumoniae subsp. pneumoniae* HS11286 (GenBank accession CP003200.1). Reference sequences are indicated as WT. Only isolates exhibiting amino acid alterations are shown, and isolates sharing identical protein sequences and amino acid alteration profiles are grouped. Altered residues are highlighted. Corresponding nucleotide substitutions are provided in Supplementary Table 1. The asterisk (*) indicates a stop codon (red highlight).
