# Supplementary material for: An integrated molecular, phenotypic, and adaptive analysis of polymyxin resistance in KPC-producing Klebsiella pneumoniae
Source: World J Microbiol Biotechnol. 2026 Nov 11;42(11):652. doi: 10.1007/s11274-026-05240-1 (PMC13754935; doi:10.1007/s11274-026-05240-1)
Supplement: Supplementary file 4 [file 11274_2026_5240_MOESM4_ESM.docx]

Supplementary Figure 1. Virulence profiles of polymyxin B-resistant *Klebsiella pneumoniae* isolates in the *Galleria mellonella* infection model.

Groups of 10 larvae were infected with bacterial suspensions adjusted to 1.5 × 10⁸ CFU/mL and monitored daily for survival for up to eight days. PBS was used as the negative injection control, and naïve larvae were included as uninjected controls. Based on the survival profiles observed under the experimental conditions, isolates were descriptively categorized as Group A, low/non-virulent phenotype (90–100% larval survival at the end of the experiment); Group B, moderate-virulence phenotype (approximately 30–60% survival); or Group C, high-virulence phenotype (complete larval mortality during the observation period).
