# Supplementary material for: An integrated molecular, phenotypic, and adaptive analysis of polymyxin resistance in KPC-producing Klebsiella pneumoniae
Source: World J Microbiol Biotechnol. 2026 Nov 11;42(11):652. doi: 10.1007/s11274-026-05240-1 (PMC13754935; doi:10.1007/s11274-026-05240-1)
Supplement: Supplementary file 5 [file 11274_2026_5240_MOESM5_ESM.docx]

Supplementary Figure 2. Short-term survival of selected *Klebsiella pneumoniae* isolates following a 1-h polymyxin B challenge under different pH conditions.

Isolates were cultured in LB adjusted to pH 5.8 or 7.7 and subsequently incubated for 1 h either without polymyxin B or with polymyxin B at 10× the previously determined MIC for each isolate. Viable cells were quantified by colony-forming unit (CFU) counting, and survival following polymyxin B exposure was evaluated relative to the corresponding culture maintained without polymyxin B at the same pH. *K. pneumoniae* ATCC 13883 was included as the experimental control.
