# Supplementary material for: An integrated molecular, phenotypic, and adaptive analysis of polymyxin resistance in KPC-producing Klebsiella pneumoniae
Source: World J Microbiol Biotechnol. 2026 Nov 11;42(11):652. doi: 10.1007/s11274-026-05240-1 (PMC13754935; doi:10.1007/s11274-026-05240-1)
Supplement: Supplementary file 6 [file 11274_2026_5240_MOESM6_ESM.docx]

Supplementary Figure 3. MALDI-TOF MS lipid A profile of the *Escherichia coli* D12 mutant recovered after polymyxin B selective pressure.

The spectrum shows lipid A-associated ions consistent with the incorporation of phosphoethanolamine (PEtN; MW, 123), 4-amino-4-deoxy-L-arabinose (L-Ara4N; MW, 131 Da), and palmitate/C16 (, MW, 238). The corresponding mass shifts are indicated in the spectrum. PEtN addition is indicated in red, L-Ara4N addition in orange, and palmitate/C16 addition in green.
