# Supplementary material for: An integrated molecular, phenotypic, and adaptive analysis of polymyxin resistance in KPC-producing Klebsiella pneumoniae
Source: World J Microbiol Biotechnol. 2026 Nov 11;42(11):652. doi: 10.1007/s11274-026-05240-1 (PMC13754935; doi:10.1007/s11274-026-05240-1)
Supplement: Supplementary file 7 [file 11274_2026_5240_MOESM7_ESM.docx]

Supplementary Figure 4. Amino acid sequence comparison of PmrA and PmrB from the *Escherichia coli* D12 mutant.

Amino acid sequences of (A) PmrA and (B) PmrB from *E. coli* D12 were compared with the corresponding sequences from *E. coli* K-12 substr. MG1655. The D12 mutant carried the PmrA substitutions S29G, I128N, and G144S and the PmrB substitutions H2R, E166A, and D282G. Nonsynonymous substitutions are highlighted in bold.
